# Vitamin K Status in Spaceflight and Ground-Based Models of Spaceflight

**DOI:** 10.1002/jbmr.289

**Published:** 2010-11-18

**Authors:** Sara R Zwart, Sarah L Booth, James W Peterson, Zuwei Wang, Scott M Smith

**Affiliations:** 1 Division of Space Life Sciences, Universities Space Research AssociationHouston, TX, USA; 2 Jean Mayer USDA Human Nutrition Center on Aging at Tufts UniversityBoston, MA, USA; 3 Enterprise Advisory Services, Inc.Houston, TX, USA; 4 Human Adaptation and Countermeasures Division, NASA Lyndon B. Johnson Space CenterHouston, TX, USA

**Keywords:** VITAMIN K, BONE LOSS, BONE TURNOVER MARKERS, SPACEFLIGHT, BED REST

## Abstract

Bone loss is a well-documented change during and after long-duration spaceflight. Many types of countermeasures to bone loss have been proposed, including vitamin K supplementation. The objective of this series of studies was to measure change in vitamin K status in response to microgravity under a variety of spaceflight and spaceflight analog (model) conditions, including long-duration spaceflight studies (*n* = 15), three bed rest studies (*n* = 15, 49, and 24), and a 14-day saturation dive (*n*= 6). In crew members who flew 2–6 months on the International Space Station, in-flight and postflight plasma phylloquinone concentrations were unchanged from the preflight mean. Consistent with this finding, urinary γ-carboxyglutamic acid (GLA), a measure of vitamin K-dependent protein turnover, did not change in response to flight. Serum undercarboxylated osteocalcin (%ucOC), a measure of vitamin K function, was generally unchanged in response to flight. Spaceflight findings were corroborated by findings of no changes in phylloquinone, urinary GLA, or %ucOC during or after bed rest in three separate bed rest studies (21–90 days in duration) or after a 14-day saturation dive. The data presented here do not support either a need for vitamin K supplementation during spaceflight or the suggestion of using vitamin K as a bone loss countermeasure in spaceflight. © 2011 American Society for Bone and Mineral Research.

## Introduction

Bone loss is a seemingly inevitable outcome of spaceflight.(1–4) Of the many types of bone loss countermeasures evaluated to date, none have been proven effective during flight.(5) Vitamin K is a nutrient linked to bone health and as such has been suggested as a potential countermeasure. Regardless of its efficacy in preventing bone loss, if the vitamin K status of astronauts were established as suboptimal during spaceflight, vitamin K supplementation might be warranted.

Vitamin K is an enzyme cofactor for the production of γ-carboxyglutamic acid (GLA) residues in specific proteins (GLA proteins). GLA proteins are involved in a number of regulatory functions, including bone mineralization. When vitamin K status is suboptimal, the assumption is that GLA proteins are not fully carboxylated and will thus be less effective. Osteocalcin is a GLA protein that is synthesized by osteoblasts and is thought to have a role in regulation of bone mineralization. Undercarboxylated osteocalcin (ucOC) provides a good measure of vitamin K functional status, with specific regard to bone.(6)

Vitamin K has been included as a concern for the nutritional status of astronauts because of uncertainty about the efficiency of vitamin K synthesis by the gastrointestinal flora in microgravity and about potentially altered absorption in this unique environment.(5) A known and consistent occurrence in the spaceflight environment is loss of bone mineral. Data showing a role for vitamin K status and/or vitamin K supplementation in bone health on Earth has led some to suggest that vitamin K treatment could play a role in protecting bone against microgravity-induced mineral loss.(7,8)

Three studies provide evidence that vitamin K status of astronauts may be suboptimal during spaceflight and suggest that vitamin K supplementation may be warranted. Carboxylation of osteocalcin was decreased in two cosmonauts during both short- and long-duration spaceflight, and it returned to preflight levels soon after landing.(9) In a case study with in-flight vitamin K supplementation, a “pharmacological dose” of 10 mg vitamin K1/day decreased undercarboxylated osteocalcin to preflight levels.(9,10) Concomitant with the positive changes in vitamin K status with supplementation, urinary calcium tended to decrease, although as the authors state, inferring a causal relation between the increased vitamin K status and the decreased urinary calcium from these data alone is highly speculative.(10)

Finally, a third observational study of plasma phylloquinone in 11 astronauts before and after 4–6 months of spaceflight on the International Space Station (ISS) reported an approximate 40% decrease in plasma phylloquinone after flight, with no change in urinary GLA excretion.(11) The estimated menu content of vitamin K in the foods provided to astronauts aboard the ISS is 105 ± 19 µg/day,(5) which is more than the documented ISS requirement (80 µg/day)(12) but approximates the median usual intake of 120 µg/day for adult males based on the Third National Health and Nutrition Examination Survey (NHANES III; 1988–1994).(13)

It is not known whether the apparent changes in vitamin K status after flight result from an effect of microgravity on vitamin K metabolism or simply from a change in dietary intake during flight. If the change in vitamin K status is a decrease with clinical relevance, then we would expect concurrent changes in the functional biomarkers of vitamin K status as well. In this paper we present data showing the effect of microgravity on markers of vitamin K status in astronauts before, during, and after long-duration flights on the International Space Station, as well as the effects of several ground analogs of spaceflight, including bed rest and saturation diving, where dietary intake was carefully monitored.

## Methods

Although the basic results (general nutritional results and results from other experiment teams, including bone, muscle, and cardiovascular physiology) from the bed rest studies reported here have been previously published, this is the first report of vitamin K status in these studies. Details and citations from earlier studies are included below.

As stated in the original publications, all protocols described below were reviewed and approved by the NASA Johnson Space Center Committee for the Protection of Human Subjects (and often by other local institutional review boards), and written informed consent was obtained from all subjects. The analysis of vitamin K in these studies was also approved by the Tufts Medical Center Institutional Review Board.

### Spaceflight

Subjects (*n* = 15, 11 male and 4 female) were astronauts on International Space Station Expeditions 14–22 (missions of 48–215 days duration, flown between 2003 and 2010). Blood and two consecutive 24-hour urine samples were collected approximately 180 and 45 days before launch and again on landing day (return, or R + 0 days). Phylloquinone and urinary GLA data from the first 11 US International Space Station crew members (Expeditions 1–8) have been published.(11) In addition to the nominal pre- and postflight collections, these crew members provided five blood and 24-hour urine collections during spaceflight at about flight day 15 (designated FD15), FD30, FD60, FD120, and FD180. Given that flight durations varied, not all crews had five in-flight sessions. Blood samples were collected using standard phlebotomy techniques. For in-flight blood collections, samples were collected into evacuated serum or plasma gel separator tubes. The tubes were centrifuged at 3800 RPM (1500 × *g*) for 30 min and then placed in a −80°C freezer on the International Space Station (with a typical temperature of −96°C). The samples were returned to Earth within 6 to 12 months in a specially designed cold bag that was capable of maintaining at least −30°C or lower until landing or in a powered −96°C freezer on the Space Shuttle. Within hours of landing, samples were transferred to dry ice for transport to Houston, where they were stored in a −80°C freezer until analysis. Except for samples collected on R + 0 (landing day), all blood samples were collected after an 8-hour fast. Urine collected before and after flight was collected into single-void urine containers (Cole-Parmer, Niles, IL, USA). Samples were stored with ice packs or refrigerated until they were processed, within 24 hours of collection. A pooled 24-hour aliquot was prepared for GLA analysis and stored frozen at −80°C until analysis. In-flight urine voids were collected into urine collection devices containing 1 mL of a LiCl solution as a volume marker. After being voided into the device, the sample was thoroughly mixed, and a syringe aliquot was obtained and then frozen until return to Earth about 6–12 months later. After landing, the in-flight urine samples were analyzed for lithium concentration to determine void volume and subsequently to prepare 24-hour pools, as previously described.(3,14)

### Bed rest

Vitamin K status of subjects in three different bed rest studies was determined. The first study was a 21-day head-down-tilt (HDT) bed rest with or without an artificial gravity (AG) countermeasure, performed at the General Clinical Research Center (GCRC) at the University of Texas Medical Branch (UTMB) in Galveston, TX, USA. The details of the AG protocol are described in other articles.(15,16) Briefly, during the bed rest phase of the study, all 15 subjects (8 AG and 7 controls, all male) were confined to 6° HDT bed rest. AG subjects were exposed to short-radius centrifuge loading for 60 min each day during bed rest to achieve z-axis loading of 2.5 G at the feet and about 1.0 G at the heart. Blood samples and 24-hour urine pools were collected for vitamin K–related analyses twice before, three times during, and twice after bed rest. Diets were controlled and were matched for all subjects by relative study day.

The second bed rest study reported here is from a series of 60- to 90-day bed rest studies with (*n* = 18, 12 male and 6 female) or without (*n* = 31, 21 male and 10 female) a vibration countermeasure on a vibration plate placed at the feet during bed rest. The details of the vibration protocol are described elsewhere.(17) The studies were performed at the UTMB GCRC, and diets were controlled and matched for all subjects. Details about the food system used for the 21-day and 60- to 90-day bed rest studies at UTMB have been published.(18,19)

A third study was a 60-day 6° HDT bed rest study with or without an exercise or nutrition countermeasure, conducted at the Institute for Space Medicine and Physiology (MEDES) in Toulouse, France. All of the subjects in this study, dubbed the “WISE” (Women's International Space Simulation for Exploration) study, were female. Details about the study protocol have been published.(20) Briefly, the control group (*n* = 8) did not exercise, nor were they provided a nutritional countermeasure during bed rest. The exercise group (*n* = 8) performed aerobic and resistive exercise during bed rest and the nutrition group (*n* = 8) was given an amino acid nutritional supplement during bed rest. Blood samples and 24-hour urine sample pools were collected twice before, three times during, and once after bed rest.

### NEEMO

Another ground analog of spaceflight in which we determined vitamin K status was the NASA Extreme Environment Mission Operations (NEEMO) V project, which was a 14-day saturation dive with crew members (*n* = 6, 2 female, 4 male) living in a small habitat about 60 m below the ocean surface, about 4 km (2.5 mi) off the coast of Key Largo, FL.(21) In this study, blood and 24-hour urine samples were collected twice before, twice during, and twice after the dive. Foods consumed during the mission were flight-like foods, and the quantities consumed were recorded and analyzed for nutrient content.(21)

### Biochemical analyses

Undercarboxylated osteocalcin from all studies presented was measured in serum using an established method(6) and a commercially available RIA kit (Biomedical Technologies, Stoughton, MA, USA). The assay uses a polyclonal antibody for intact human osteocalcin and recognizes both intact osteocalcin and the large N-terminal midmolecule fragment.(6) Undercarboxylated osteocalcin analyses for all studies reported, except spaceflight, were run by Dr. Booth's laboratory; analyses for the spaceflight study were run at the Johnson Space Center in Houston, TX.

In blood samples from all subjects in the artificial gravity bed rest study, the 60-day bed rest study in France, and the NEEMO V study, and from 18 subjects in the 60- to 90-day bed rest study at UTMB, plasma phylloquinone was measured at Tufts University using a liquid-chromatographic method with fluorometric detection.(22) In all samples from the 15 crew members with pre-, in-, and postflight data and in samples from 31 subjects in the 60- to 90-day bed rest studies, plasma phylloquinone was measured in the Nutritional Biochemistry Laboratory at the NASA Johnson Space Center (JSC) using the microelution solid-phase extraction/HPLC method described below. Both labs participate in the vitamin K external quality assurance scheme (KEQAS) that monitors and reports the accuracy of phylloquinone measurements. KEQAS samples from lots 41, 42, and 43 were measured in both labs and the coefficient of variation was determined to be 13 ± 5%.

Urinary GLA from the artificial gravity bed rest study, the 60-day bed rest study in France, the NEEMO V study, and some of the spaceflights was determined by reversed-phase HPLC with fluorometric detection(23) at Tufts University. The remaining GLA samples from spaceflight and the 60- to 90-day bed rest study were quantified using a precolumn derivatization with ortho-phthaldialdehyde and a silica-based anion-exchange HPLC column(11) in the Nutritional Biochemistry Laboratory at JSC.

Plasma phylloquinone was measured at JSC using microelution solid-phase extraction. Separation was performed using an Agilent (Santa Clara, CA, USA) 1100 HPLC system equipped with a Thermo Scientific (West Palm Beach, FL, USA) Hypersil-100 C18 column (4.6 × 150 mm, 5 µm particle size), using postcolumn reduction and fluorescence detection with the excitation wavelength at 244 nm and emission at 430 nm. The mobile phase contained, per liter, 994.5 mL methanol and 5.5 mL of an aqueous solution of 2 mol/L zinc chloride, 1 mol/L acetic acid, and 1 mol/L sodium acetate. An isocratic mode with a flow rate of 1.5 mL/min was used in the assay. The column temperature was maintained at 30°C during analysis. Plasma samples were precipitated by 1:4 (v/v) dilution with ethanol after adding 20 µL of 1:19 (v/v) ethanol-diluted internal standard (a proprietary vitamin derivative that is a component of a commercial assay kit for vitamin K1 assays, ALPCO Diagnostics, Salem, NH, USA) and then centrifuged at 13,500 RPM (18,779 × *g*) at 4°C for 5 min. The Waters Oasis HLB 96-well microelution solid-phase extraction plate (Waters Corp., Milford, MA, USA) was equilibrated with 0.25 mL methanol followed by 0.25 mL deionized water. Then 0.1 mL deionized water was added to the plasma precipitate, and the supernatant of the sample was transferred to the microextraction plate. The plate was washed with 0.25 mL 50% methanol in deionized water and then eluted with 75 µL of 1:1 isopropanol:acetonitrile (v/v) into a Waters 0.8-mL round well, 96-well collection plate (Waters Corp.). The sample was transferred to a 400-µL flat-bottom glass insert (Alltech, Deerfield, IL, USA) in a 1.5-mL amber vial and capped; 60 µL was used for HPLC analysis.

### Statistical analyses

A repeated-measures one-way ANOVA was performed on spaceflight data and NEEMO data. A repeated-measures two-way ANOVA was performed on the bed rest studies, with time and countermeasure (exercise and artificial gravity) as variables. Each bed rest study was analyzed separately. A separate repeated-measures two-way ANOVA was performed to determine whether the lab where analyses were performed had an effect on vitamin K values. If overall significant differences were detected, a post hoc Bonferroni *t*-test was performed to establish differences between time points or within treatments. Statistical analyses were performed using Sigma Stat 3.11 (Systat Software, San Jose, CA, USA).

## Results

### Spaceflight

The phylloquinone data from the 15 subjects with pre-, in-, and postflight data are presented in Table 1. There was one outlier at FD60 (the value was 13.2 nmol/liter). Only when the outlier was removed, the phylloquinone concentration at FD15 was significantly less than the preflight mean, but the preflight mean was not different from the other time points.

**Table 1:** Markers of Vitamin K Status Before, During, and After Long-Duration Spaceflight on ISS

	Preflight	FD15	FD30	FD60	FD120	FD180	R + 0	R + 30
Phylloquinone (nmol/L)	1.4 ± 0.7	0.4 ± 0.3	1.3 ± 1.2	1.7 ± 3.2	0.7 ± 0.3	0.7 ± 0.5	1.0 ± 0.6	0.9 ± 0.8
*n*	15	15	15	15	12	9	15	15
GLA (µmol/day)	38 ± 7	41 ± 14	40 ± 9	40 ± 9	40 ± 10	46 ± 12	40 ± 7	45 ± 9
*n*	15	15	15	15	12	9	15	15
ucOC (%)	37 ± 7	43 ± 6*	39 ± 4	40 ± 5	41 ± 5	41 ± 7	42 ± 6*	35 ± 5
*n*	14	13	12	14	11	8	15	15

Data are means ± SD. FD, flight day; R + 0, return + 0 days (landing day); GLA, γ-carboxyglutamic acid; ucOC, undercarboxylated osteocalcin. Phylloquinone was measured in plasma, GLA in urine, and ucOC in serum. The data reported above include all available data. There was one outlier in the phylloquinone data at FD60 (value was 13.2 nmol/L); when the outlier was removed, the remaining data were 0.9 ± 0.7, and there was a significant decrease in phylloquinone at FD15 (*p* < .01). The data were analyzed using a repeated-measures one-way ANOVA, and there were no significant differences when the one outlier was not removed.

* *p* < .001, significantly different from preflight.

The postflight (R + 0) mean of urinary GLA was not different from the preflight mean, nor was there a difference during flight (Table 1).

Undercarboxylated osteocalcin was significantly higher on FD15 and R + 0, but these changes were small, and likely reflect the narrow range of the preflight data (Table 1). Although a dramatic increase seemed to occur at FD180, this happened largely because the number of subjects on flights of this duration (and thus with data available at this time point) was very small.

Unfortunately, dietary phylloquinone intake data were not available from the flight studies at any time before, during, or after flight.

### Bed rest

Average consumption of vitamin K before, during, and after bed rest for the AG group in the 21-day bed rest study was 174 ± 39 (mean ± SD), 138 ± 29, and 172 ± 38 µg/day and for the control group was 207 ± 14, 161 ± 15, and 202 ± 15 µg/day, respectively. The mean vitamin K intake before, during, and after bed rest for the controls in the 60- to 90-day bed rest studies was 137 ± 26, 120 ± 24, and 134 ± 25 µg/day, respectively. For the subjects with the vibration countermeasure, vitamin K intake before, during, and after bed rest was 158 ± 26, 128 ± 17, and 131 ± 24 µg/day, respectively. Dietary phylloquinone intake data were not available from the 60-day study conducted in France.

No significant differences in plasma phylloquinone concentration were found during or after 21 days of bed rest with or without artificial gravity (Table 2), nor was there a difference after 60 days of bed rest with or without exercise or a nutritional countermeasure (Table 3). With or without a vibration countermeasure, plasma phylloquinone concentration decreased 35–38% in 8 days after reambulation from 60–90 days of bed rest (*p* < .01, Table 4). There was no significant difference between analyses performed at the Johnson Space Center and those performed at Tufts. Urinary GLA was significantly affected by time during the 21-day (Table 2) and 60- to 90-day (Table 4) bed rest studies, where a significant main effect occurred, but there were no differences between time points when the post hoc test was performed. Undercarboxylated osteocalcin was not different during bed rest in any of the bed rest studies, but in the vibration group in the 60- to 90-day bed rest studies, it was lower overall than in controls (Table 4, *p* < .01).

**Table 2:** Vitamin K Status Before, During, and After 21 Days of Head-Down-Tilt Bed Rest in Men With or Without an Artificial Gravity Countermeasure

	Pre-BR	BR8	BR15	BR21	R + 0/1	R + 8	*n*
Phylloquinone (nmol/L)							
AG	2.2 ± 0.9	1.9 ± 0.8	1.8 ± 0.7	1.7 ± 0.6	N/A	1.9 ± 0.8	8
Control	2.7 ± 0.9	3.1 ± 0.9	2.8 ± 1.2	2.8 ± 1.2	N/A	2.3 ± 1.1	7
GLA^a^ (µmol/day)							
AG	47 ± 13	48 ± 14	45 ± 14	46 ± 12	43 ± 14	48 ± 14	8
Control	50 ± 14	49 ± 11	50 ± 11	48 ± 10	47 ± 11	51 ± 11	7
ucOC (%)							
AG	46 ± 18	43 ± 23	34 ± 15	39 ± 26	N/A	27 ± 16	8
Control	39 ± 14	34 ± 16	42 ± 14	32 ± 11	N/A	39 ± 7	7

Data are means ± SD. BR8, bed rest day 8; R + 0/1, recovery + 0 or 1 day after reambulation; AG, artificial gravity countermeasure (centrifugation while in head-down-tilt position to achieve z-axis loading of 2.5 G at the feet and 1.0 G at the heart for 1 h per day for 21 days). N/A, data not available; GLA, γ-carboxyglutamic acid; ucOC, undercarboxylated osteocalcin. Phylloquinone was measured in plasma, GLA in urine, and ucOC in serum. Data were analyzed using a repeated-measures two-way ANOVA.

a Significant effect of time, *p* < .05 (no differences between time points with a post hoc Bonferroni *t*-test).

**Table 3:** Vitamin K Status Before, During, and After a 60-Day Head-Down-Tilt Bed Rest in Women With or Without an Exercise or Nutrition Countermeasure

	Pre-BR	BR15	BR30	BR59	Post-BR
Phylloquinone (nmol/L)					
Exercise	1.0 ± 0.4	N/A	1.7 ± 0.8	1.5 ± 0.7	1.3 ± 0.7
Control	1.1 ± 0.6	N/A	1.3 ± 0.6	1.2 ± 0.7	0.9 ± 0.4
Nutrition	1.3 ± 0.6	N/A	0.9 ± 0.2	1.0 ± 0.3	1.0 ± 0.5
GLA (µmol/day)					
Exercise	39 ± 8	40 ± 6	38 ± 6	35 ± 19	42 ± 18
Control	36 ± 7	34 ± 8	35 ± 11	34 ± 6	38 ± 9
Nutrition	35 ± 3	36 ± 3	37 ± 6	35 ± 5	34 ± 5
ucOC (%)					
Exercise	39 ± 10	N/A	41 ± 7	43 ± 17	45 ± 20
Control	33 ± 15	N/A	41 ± 13	41 ± 13	42 ± 7
Nutrition	43 ± 7	N/A	44 ± 9	42 ± 15	40 ± 15
*n*	8	8	8	8	8

Data are means ± SD. BR, bed rest; post-BR, blood or urine collected between 5 and 7 days after reambulation; GLA, γ-carboxyglutamic acid; N/A, data not available; ucOC, undercarboxylated osteocalcin. The data were analyzed by repeated-measures two-way ANOVA and there were no significant differences.

**Table 4:** Vitamin K Status Before, During, and After 60–90 Days of Head-Down-Tilt Bed Rest With or Without a Vibration Countermeasure

	Pre-BR	BR30	BR60	BR90	R + 8	*n*
Phylloquinone^a^ (nmol/L)						
Control	1.7 ± 1.0	1.4 ± 1.3*	1.5 ± 1.4	1.5 ± 1.2	1.1 ± 0.8*	31
Vibration	2.1 ± 1.4	1.5 ± 0.9	2.0 ± 1.3	1.7 ± 1.0	1.3 ± 0.6*	18
GLA (µmol/day)						
Control	40 ± 9	41 ± 10	36 ± 11	41 ± 8	42 ± 13	31
Vibration	41 ± 11	41 ± 10	43 ± 12	42 ± 11	42 ± 13	18
ucOC (%)						
Control	48 ± 17	46 ± 21	56 ± 13	50 ± 14	52 ± 17	31
Vibration^b^	26 ± 21	40 ± 26	40 ± 33	32 ± 21	38 ± 31	18

Data are means ± SD. BR, bed rest; R + 8, 8 days after reambulation from bed rest; GLA, γ-carboxyglutamic acid; ucOC, undercarboxylated osteocalcin.

a Significant effect of time, *p* < .01;

b significant group effect, *p* < .01.

* Significantly different from pre-BR as determined by a post hoc Bonferroni *t*-test.

### NEEMO

When crew members on a 14-day saturation dive consumed spaceflight-like foods containing a per-subject total of 23 ± 14 µg vitamin K/day during the dive, neither plasma phylloquinone nor urinary GLA concentration changed over the course of the mission (Table 5).

**Table 5:** Vitamin K Status Before, During, and After a 14-Day Saturation Dive

	Pre-dive	MD7	MD12	R + 0	R + 1	R + 7	R + 8
Phylloquinone (nmol/L)	1.5 ± 1.0	2.0 ± 3.1	1.0 ± 0.6	0.7 ± 0.5	N/A	N/A	2.5 ± 1.8
GLA (µmol/day)	38 ± 7	37 ± 11	32 ± 10	43 ± 29	36 ± 12	42 ± 11	37 ± 10
*n*	6	6	6	6	6	6	6

Data are means ± SD. MD, mission day; R + 0, return plus 0 days; GLA, γ-carboxyglutamic acid; N/A, data not available. The data were analyzed by one-way ANOVA and no significant differences were found. Blood samples were not collected on days R + 1 or R + 7.

## Discussion

The limited spaceflight data available related to vitamin K led investigators to propose that vitamin K status was altered during long-duration spaceflight. Data from the first 11 US crew members aboard the International Space Station (4–6 months of spaceflight) showed a 42% decrease in plasma phylloquinone concentration after spaceflight.(11) Two cosmonauts on the Russian space station Mir had a rapid increase in undercarboxylated osteocalcin that remained high during a 21-day and a 180-day spaceflight.(9) One of those two cosmonauts was also the subject in a case study in which the cosmonaut's carboxylated osteocalcin increased after vitamin K supplementation during the 180-day flight.(9,10) Taken together, these lines of evidence suggest that vitamin K status is lower during flight. However, as reported here, in plasma samples from 15 crew members who have flown on the ISS since 2006, we found no change in plasma phylloquinone concentration during or after flight compared to the mean preflight concentration. More importantly, however, for the first time we have analyzed plasma phylloquinone, serum undercarboxylated osteocalcin, and urinary GLA during flight, and the data presented here do not support the idea that a decrease in vitamin K status is induced by microgravity.

Plasma phylloquinone decreased early during flight, but after 180 days of spaceflight its concentration was not different from preflight concentrations (Table 1). Because we observed a decrease in vitamin K status in our previous study,(11) we would have expected to see an increase in undercarboxylated osteocalcin and decrease in urinary GLA, and neither was observed during or after long-duration spaceflight. Fluid shifts might account for some of this (through increased plasma volume and dilution of phylloquinone), but a general decrease in plasma constituents was not observed after flight (although this may be related to tighter control over other constituents). Another explanation is related to phylloquinone intake. It is well documented that plasma phylloquinone concentrations fluctuate within 24 hours in response to changes in dietary phylloquinone intakes.(23–25) Dietary intake in general was likely lower in the last 24 hours of flight, and this could explain the decreased plasma phylloquinone on landing day in our previous study.(11) Without a concomitant decline in vitamin K status as measured by functional biomarkers, the transient decrease in phylloquinone on landing day is likely not clinically relevant. Furthermore, in samples collected 30 days after landing, when fluid balance would have largely returned toward normal, patterns observed in the data were similar to those on landing day.

Aside from any association with spaceflight, a low vitamin K status is often associated with lower bone mass in observational studies.(26) However, the preponderance of randomized clinical trials in men and women suggest that phylloquinone supplementation has no impact on age-related bone loss under conditions of normal gravity (that is, on the ground).(27) One suggestion for the discrepancy between the findings of observational studies and the clinical trials is that vitamin K intake tracks a healthy lifestyle, and hence there is lower risk for bone loss.(28,29) This inconclusiveness of the ground-based data on phylloquinone supplementation for bone protection further supports the idea that supplementation during spaceflight for mitigation of bone loss is not warranted at this time. The vitamin K supplementation case study was conducted on the Russian space station Mir in 1995, and the food system was composed entirely of Russian foods. On the ISS, and on Mir flights when US crew members were aboard, the food system was about half American space food and half Russian space food. Whether this difference affected vitamin K intakes is impossible to know, but the supplement administered in the Russian space study contained 10 mg/day, which, given its high dose, would have superseded any dietary effects on measures of vitamin K status.

Although the initial goals and hypotheses of the bed rest studies presented here did not include the study of vitamin K status in particular, we presented the data here as evidence supporting the spaceflight data. Bed rest is a well-accepted model for certain aspects of spaceflight.(18,30,31) No striking changes occurred in phylloquinone, urinary GLA, or undercarboxylated osteocalcin during bed rest in any of the studies, nor was vitamin K status affected by any of several countermeasures.

The data presented here, though largely negative, are important nonetheless. This is the first complete picture of a vitamin K status assessment during spaceflight with supporting data from ground-based models of spaceflight. The data presented here do not support vitamin K supplementation as a countermeasure during spaceflight to prevent a vitamin K deficiency (or mitigate bone loss), because there was no evidence for a decrease in vitamin K status during flight.
